# An Investigation on the Therapeutic Effect of Thymosin β4 and Its Expression Levels in Streptozotocin-Induced Diabetic Mice

**DOI:** 10.1155/2018/3421568

**Published:** 2018-08-27

**Authors:** Kyung Sook Cho, Dong-Jin Kim, Bomee Shim, Jung Yeon Kim, Jun Mo Kang, Seon Hwa Park, Sang-Ho Lee, Hyung-In Yang, Kyoung Soo Kim

**Affiliations:** ^1^Department of Clinical Pharmacology and Therapeutics, College of Medicine, Kyung Hee University, Seoul, Republic of Korea; ^2^Division of Nephrology, Department of Internal Medicine, Kyung Hee University Hospital at Gangdong, Kyung Hee University, Seoul, Republic of Korea; ^3^Department of Pathology, Inje University Sanggye Paik Hospital, Nowon-gu, Seoul, Republic of Korea; ^4^Division of Nephrology, Department of Internal Medicine, CHA Bundang Medical Center, CHA University, Seongnam, Republic of Korea; ^5^Division of Rheumatology, Department of Internal Medicine, Kyung Hee University Hospital at Gangdong, Gangdong-gu, Seoul, Republic of Korea; ^6^East-West Bone & Joint Disease Research Institute, Kyung Hee University Hospital at Gangdong, Gandong-gu, Seoul, Republic of Korea

## Abstract

Thymosin *β*4 (T*β*4) treatment was known to show the potential therapeutic effects on diabetic complications. This study was performed to determine if T*β*4 expression is changed in both serum and tissues under diabetic conditions and can be a serum biomarker. Type 1 diabetic mice were induced in C57/BL6J mice by intraperitoneal injection of streptozotocin (STZ) at a dose of 50 mg/kg body weight. The mice were sacrificed at 16 weeks after STZ injection. Tissues and plasmas were obtained to determine the expression levels of T*β*4 using ELISA, real time RT-PCR, and immunohistochemistry. The average serum glucose level was increased to approximately 400 mg/dL beginning 2 weeks after the five injections of STZ and lasting for at least 13 weeks until sacrifice. The plasma and tissue levels of T*β*4 in the age-matched control mice were not significantly different from those of the diabetic mice. In conclusion, the T*β*4 expression level in the plasmas and tissues of diabetic mice was not affected by diabetic conditions. It indirectly suggests that the therapeutic effect of T*β*4 on diabetic complications is due to its regenerative effects on damaged tissue but not to the changed expression level of T*β*4 in plasma and tissues of diabetes.

## 1. Introduction

Thymosin *β*4 (T*β*4), a 4.9 kDa peptide with 43 amino acids, has a number of active sites. The T*β*4 containing amino acids 1-4 has anti-inflammatory activity, residues 1-15 have anti-apoptotic and cytoprotective activity, and residues 17-23 affect cell migration, actin biding, dermal wound healing, angiogenesis, and hair growth [1]. Specifically, it binds to G-actin to regulate the formation of F-actin, which in turn regulates the dynamics of cytoskeletal rearrangement [2], leading to effects on cell mobility, cell division, and differentiation. T*β*4 is secreted into bodily fluids at a relatively high concentration even though there are no known signals for its secretion, and it has numerous physiological and pathological effects on cells and organs although it has no known receptors. For instance, it aids in wound healing [3], tumor metastasis, angiogenesis [4], cell migration [5], and stem cell differentiation [6]. Because increased T*β*4 expression may worsen clinical outcomes from tumors [7], T*β*4 is a potential molecular target for tumor treatment [8]. In contrast, decreased levels of T*β*4 in the kidneys accelerated glomerular disease in global T*β*4 knockout mice [9]. In addition, T*β*4 treatment has been used to alleviate the symptoms of various diseases of animal models. Thus, T*β*4 serum level has been measured for developing potential biomarker in various diseases such as cardiovascular, infectious, and autoimmune disease [10].

Treatment with T*β*4 or its active fragment, N-acetyl-seryl-aspartyl-lysyl-proline (Ac-SDKP), helps to recover function following a brain injury by inhibiting the TGF-*β*1/NF-*κ*B signaling pathway [11]. It also aids in dermal healing because of its ability to stimulate tissue repair and regeneration[12]. Thus, T*β*4 therapeutic treatments have been tested in phase 2 clinical trials of patients with pressure ulcers, stasis ulcers, and epidermolysis bullosa wounds [12]. In addition, the therapeutic effects of T*β*4 have been widely studied in the field of diabetes mellitus. In particular, T*β*4 treatment showed therapeutic effects on diabetic peripheral nephropathy [13] and neuropathy [14, 15] in diabetic mice. Coadministration of thymosin fraction 5 (TF5), which contains T*β*4 [16], with a subdiabetogenic dose of streptozotocin (STZ, 35 mg/kg) blocked the initiation of insulitis and hyperglycemia in mice [17]. A high dose of TF5 (0.1 mg/day) maintained normal blood glucose levels in diabetic mice, but treatment with low-dose TF5 (0.01 mg/kg) had no effect on blood glucose levels. The protective effects of T*β*4 could be partly induced by modulating the immune-regulatory or immunostimulating effects on suppressor T cells [18].

Despite extensive studies on T*β*4 treatment and the potential therapeutic effects of T*β*4 on diabetic complications, the blood and tissue expression levels of T*β*4 during the progression of diabetes mellitus have not been thoroughly investigated. In this study, we determined if T*β*4 expression is changed in both plasma and tissues under diabetic conditions using an STZ-induced type 1 diabetic mouse model.

## 2. Materials and Methods

### 2.1. Cell Culture of Isolated Splenocytes

Splenocytes were isolated from the spleens of C57/BL6J mice as described previously [19]. The splenocytes were seeded at 3 × 10^6^ cells/well in 6-well plates. The cells were stimulated with a high concentration of glucose (20-60 mM) in 2 ml of DMEM for 24 hrs. The culture supernatants were harvested for the analysis of T*β*4 protein levels using ELISAs. Total RNA was extracted from the cells for the analysis of T*β*4 mRNA levels using real time PCR.

### 2.2. Preparation of Diabetic Mice

Diabetes was induced in 8-week-old male C57/BL6J mice as described previously [20]. Briefly, C57BL6J mice (*n=9*) were intraperitoneally injected with streptozotocin (STZ) (50 mg/kg/day), which was dissolved in 50 mM Na citrate buffer (pH 4.5), for 5 consecutive days. Blood glucose levels were checked every 7 days using a blood glucose meter (G-Doctor, Allmedicus, Anyang, Korea) and blood from the tail, and body weights were also measured weekly. Mice with blood glucose levels less than 250 mg/dL at 2 weeks after the final injection of STZ were excluded from the experiment. The mice were sacrificed 16 weeks after the final administration of STZ, and an age-matched control group (*n=6*) was also sacrificed at the same time. Mice were anesthetized with the mixture of Zoletil (30mg/kg) and xylazine (10mg/kg). The blood was collected from the inferior vena cava into heparinized syringes. Before collecting the blood, heparin (5000IU/ml, JW-Pharma, Seoul, Korea) was sucked into a syringe and the syringe was moved repeatedly up and down to coat the heparin. Then heparin was removed from the syringe. After blood collection, remained blood was removed through cardiac perfusion with cold saline. The blood was centrifuged (2000xg, 20 minutes, 4°C) and plasma was collected and stored in deep-freezer before analysis. Also, the organs from both groups were obtained. All the samples were kept at −80°C or in 10% buffered formalin for mRNA and immunochemistry analysis. Two separate replicate experiments were done, and all experiments were performed with approval (KHNMC AP 2016-007) according to the guidelines of the Gangdong Animal Research Ethics Committee of Kyung Hee University Hospital.

### 2.3. Real Time Polymerase Chain Reaction (PCR)

Total RNA was extracted from splenocytes and the tissue of each organ using Trizol (Thermo Fisher Scientific Korea, Seoul). The cDNA of the RNA was synthesized using a commercial cDNA synthesis kit (Thermo Fisher Scientific Korea, Seoul) according to the manufacturer's instructions. Real time PCR was performed using SYBR Green PCR mater mix (Applied Biosystems, Carlsbad, CA, USA) and the following primer sequences: T*β*4-forward 5′-CTC GGC TCC TTC CAG CAA C-3′, T*β*4-reverse 5′-TCG CCA GCT TGC TTC TCT TG-3′, 18S-forward 5′-GTA ACC CGT TGA ACC CCA TT-3′, and 18S-reverse 5′-CCA TCC AAT CGG TAG TAG CG -3′.

### 2.4. T*β*4 ELISA

The T*β*4 protein levels in the plasma and the cell culture supernatants were measured using a commercial ELISA kit from Peninsula Laboratories (San Carlos, CA, USA). The ELISA was performed according to the manufacturer's instructions. The intra- and interassay coefficients of variation (CV) are less than 10% and 15%, respectively.

### 2.5. Histopathological Analysis and Immunohistochemistry

We harvested the internal organs (i.e., the stomach, intestines, pancreas, liver, spleen, kidney, thymus, lungs, and heart) from diabetic mice (*n=5*) and control mice (*n=3*). The organs were dissected, fixed with 10% buffered formalin, embedded in paraffin, and prepared into 4 *μ*m thick sections. The sections were either stained with hematoxylin and eosin (H&E) or used for immunohistochemical staining in an automated system (Leica Biosystems, Newcastle, UK) using anti-T*β*4 antibodies (Peninsula Laboratories International Inc. San Carlos, CA). The antigens were retrieved with epitope retrieval solution 1 (Leica Biosystems). The slides were incubated with each antibody at room temperature for 20 minutes then with a biotinylated secondary antibody for 8 minutes. The resulting complexes were detected using avidin-peroxidase conjugate polymer (Leica Biosystems), and the color was developed using 3,3′-diaminobenzidine (Leica Biosystems). Mayer's hematoxylin (Leica Biosystems) was used as a counterstain. Positive- and negative-control staining was also used for each assay run.

### 2.6. Statistical Analysis

GraphPad Prism software, version 5 (San Diego, CA), was used for the statistical analysis and graphing. The two-tailed Mann–Whitney test was used to compare the T*β*4 serum levels between the diabetic and control mice. The T*β*4 mRNA expression levels from triplicate tissue samples from control and diabetic mice were assessed using the Mann–Whitney test.* P* values <0.05 were considered to be statistically significant. All data are expressed as the mean ± standard error of the mean (SEM).

## 3. Results

### 3.1. Comparison of Thymosin *β*4 (T*β*4) Levels in the Plasma of Diabetic and Control Mice

**Figure 1:**
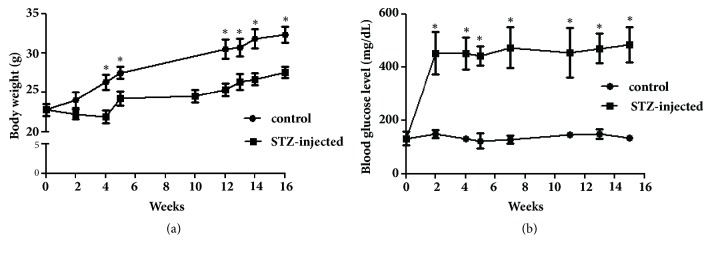
Body weights and blood glucose levels of control and streptozotocin- (STZ-) induced diabetic mice. C57BL6J mice (n=9) were intraperitoneally injected with STZ (50 mg/kg/day) for 5 consecutive days. The body weight (a) and blood glucose level (b) were measured every 7 days and compared with the control group (n=6). There were significant differences between the two groups. *∗P*<0.05.

**Figure 2:**
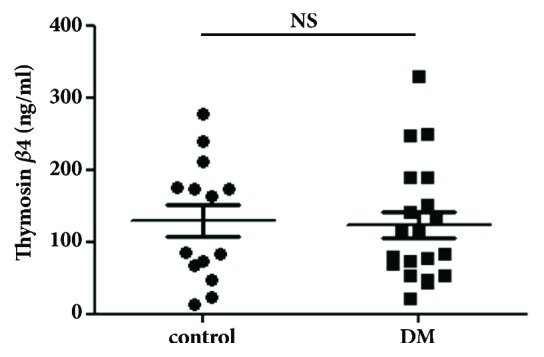
Comparison of thymosin *β*4 (T*β*4) levels in the plasma of streptozotocin-induced diabetic mice and control mice. STZ-induced type 1 diabetic mice and age-matched control mice were sacrificed at 16 weeks after the final injection of STZ. The plasma T*β*4 levels were measured using ELISA, and no significant difference was found between the two groups. NS: no significant difference; DM: diabetes mellitus.

To determine how hyperglycemia affects the expression level of T*β*4 in plasma and tissues, test mice received five consecutive injections of STZ (50 mg/kg) and were maintained for 16 weeks without any therapeutic treatments. The body weights were significantly lower in the STZ-injected mice compared to the control mice as shown in Figure 1(a). The blood glucose levels (> 400 mg/dL) in the STZ-injected mice remained significantly higher than those in the control mice beginning 2 weeks after the final STZ injection (Figure 1(b)) until sacrifice at week 16. However, the T*β*4 plasma expression levels were not significantly different between the diabetic mice (129.3 ± 22.2 ng/mL) and the control mice (123.3 ± 18.2 ng/mL) (Figure 2). This suggests that the expression level of T*β*4 is not changed in plasma of STZ-induced type 1 diabetic mice.

### 3.2. Comparison of T*β*4 Levels in the Tissues of Diabetic and Control Mice

**Figure 3:**
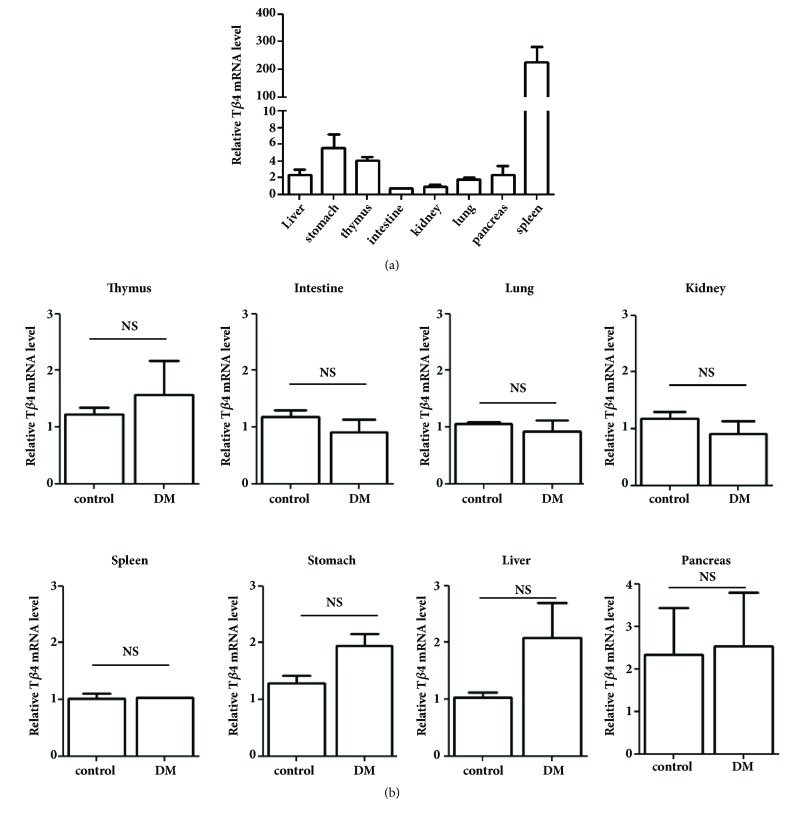
Comparison of T*β*4 mRNA levels in the tissues of diabetic and control mice. STZ-induced type 1 diabetic mice and age-matched control mice were sacrificed at 16 weeks after the final injection of STZ. Each organ was harvested and the total RNA was extracted using Trizol. The T*β*4 transcriptional level was measured using real time PCR as described in the Materials and Methods. (a) In the control mice, the spleen had the highest expression level of T*β*4 among the various organs in relative to liver. A similar pattern was observed in the diabetic mice. The data represent triplicate samples of each organ of the control mice. (b) The T*β*4 expression level of each organ of the control mice was compared with the diabetic mice. There was no significant difference in T*β*4 expression between the control and diabetic mice. The representative results are shown. NS: not significant.

**Figure 4:**
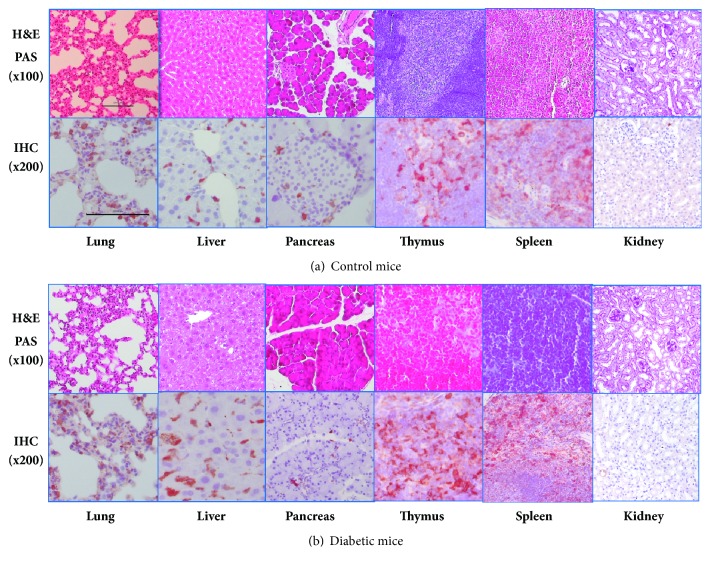
Comparison of T*β*4 protein levels in the tissues of diabetic and control mice. The difference in T*β*4 expression level and the degree of tissue damage between the two groups was compared using (a) H&E staining (×100) and (b) immunohistochemistry (×200) (scale bar, 100 *μ*m). The spleen had a higher expression level than the other tissues comparable with the difference in transcription levels. There were no significant differences between the two groups in T*β*4 protein expression level or degree of tissue damage.

Next, the relative T*β*4 mRNA expression levels were measured in various tissues such as the liver, stomach, thymus, spleen, and kidneys. The mRNA expression level in the spleen was approximately 20-fold higher than in the other tissues (Figure 3(a)). Also, the T*β*4 mRNA expression level was not affected in each of the tissues of the diabetic mice (Figure 3(b)). To further study T*β*4 expression at the protein level, tissues samples from the diabetic and control mice were compared using histology and immunohistochemistry. The histology assay of the diabetic mice showed a higher degree of damage or loss of islet cells in the pancreas and mild lobular disarray in the liver compared to the control mice (Figure 4(a)). The other tissues were not significantly different between the two groups in terms of damage or loss secondary to hyperglycemia. For immunohistochemical staining, cells of moderate or strong nuclear and/or cytoplasmic staining were determined as a percentage and scored as follows: 0 (staining in less than 10% of cells); 1 (staining in 10-50% of cells); and 2 (staining in more than 50% of cells). Cases with score 1 or 2 were classified as positive. T*β*4 staining was positive for the lungs, liver, thymus, spleen, stomach, and small intestine (score 1), but not in the heart and pancreas (score 0) (Figure 4(b)), and revealed no difference in T*β*4 expression between the diabetic and control mice. Specifically, the lung samples had T*β*4-positive staining cells in the interstitial tissue of both the diabetic and the control mice. The Kupffer cells and sinusoidal cells in the liver were stained for T*β*4 in both the diabetic and control mice, but the number of positive-stained cells was higher in the diabetic mice than in the control mice. The pancreas showed a few positive-stained cells only in the diabetic mice (score 0). The thymus revealed scattered T*β*4-positive staining in both the cortex and the medulla, and T*β*4-positive cells were noted in both the white pulp and the red pulp of the spleen. Taken together, these results indirectly suggest that diabetic conditions such as hyperglycemia do not significantly affect the serum and tissue levels of T*β*4 in type 1 diabetic mice.

### 3.3. Effects of High-Glucose Concentrations on the Expression of T*β*4 in Splenocytes* In Vitro*

**Figure 5:**
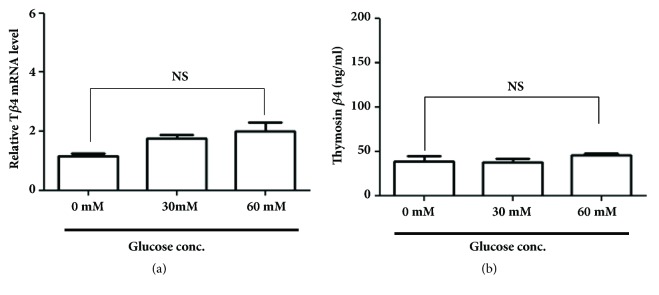
Effects of glucose concentration on the* in vitro* expression of T*β*4 in splenocytes. Immune cells from the spleen were isolated and treated with high-glucose concentrations for 24 hrs. Subsequently, the total RNA was extracted from the cells for real time PCR analysis (a) and the cell culture supernatant was obtained to determine the T*β*4 levels using ELISA (b). Three independent experiments were performed using triplicates of each sample for the real time PCR and ELISA. Similar results were obtained from each experiment, and representative results are shown. NS: not significant.

To determine how high-glucose concentration affects the* in vitro* expression of T*β*4 in immune cells that highly express T*β*4, splenocytes were treated with high concentrations of glucose for 24 hrs. The mRNA expression level of T*β*4 did not significantly increase in the glucose-treated group compared to the control group. The protein level of T*β*4 also was not increased by the high-glucose treatment (Figure 5). This suggests that high-glucose concentrations did not affect the* in vitro* expression of T*β*4 in immune cells that had a high basal level of T*β*4 expression.

## 4. Discussion

In this study, the expression levels of T*β*4 in plasma and tissues from STZ-induced diabetic mice were investigated to know if its expression could be changed in diabetic conditions. This can indirectly explain if the therapeutic effects of T*β*4 on diabetic complications could result from ameliorating the decreased expression of T*β*4. The STZ-induced type 1 diabetic mice were under hyperglycemic conditions for at least 13 weeks because they were not treated with any blood glucose lowering agents and were sacrificed 16 weeks after the final injection of STZ. Using real time PCR, ELISA, and immunohistochemistry, we determined that the expression of T*β*4 in the plasma and tissues of the diabetic mice was not significantly different than in the age-matched control mice. Marked tissue damage was also not observed in the diabetic mice, except for that in the pancreas. Taken together, these data suggest that hyperglycemia was not a factor that had some impact on T*β*4 plasma level in this model, but the T*β*4 released from damaged tissues was not able to affect the overall T*β*4 serum level.

The T*β*4 serum level has been documented to change significantly according to disease state. In our previous studies, T*β*4 level was significantly increased in the serum and synovial fluid of patients with rheumatoid arthritis [21, 22]. The T*β*4 level was significantly increased in the serum of patients with inflammatory bowel disease [23], and the level was high in human wounds and blister fluid after dermal injury [24]. This increase in T*β*4 may be due to tissue damage or cell lysis, or it may be increased by a host defense system due to T*β*4's anti-inflammatory effects [25, 26]. However, a conflicting study showed that the serum level of T*β*4 was significantly decreased in patients with liver failure caused by chronic hepatitis B virus (HBV) [27]. In diabetic mice in the current study, serum T*β*4 level was not increased or decreased compared to that of control mice. This suggests that blood glucose level did not affect T*β*4 serum level and that T*β*4 was not released into serum from damaged cells or cell lysis. Chronic hyperglycemia also induces an inflammatory state [28, 29]. Nonenzymatically glycosylated proteins interact with their receptors to induce oxidative stress and proinflammatory responses. Hyperglycemia also enhances inflammation by promoting cytokine secretion in monocytes, adipocytes, and other cells. The hyperglycemia-induced inflammation produced in this study may not have been sufficient to induce the expression of T*β*4 or the release of T*β*4 from cells through cell damage or lysis. Furthermore, we may consider the possibility that T*β*4 level might decrease (or increase) during the initial stage and gradually return to baseline as hyperglycemia continues to develop. In addition, a great amount of T*β*4 is released from platelet at sites of injury for wound healing. The serum level can be influenced by various factors [30]. However, we do not provide a detailed temporal profiling on the expression of this peptide.

A radioimmunoassay for T*β*4 demonstrated that it was ubiquitously present in most rat and mouse tissues although the spleen had higher concentrations than other tissues such as the brain, kidneys, liver, and testes [31]. In this study, the effects of hyperglycemia on T*β*4 expression in tissues were investigated using quantitative real time PCR and immunohistochemistry. As with the serum expression, T*β*4 tissue expression was not affected by hyperglycemia. Among the organ tissues of the control mice, the spleen had the highest transcriptional expression, which was comparable with the previous report [9]. The heart and pancreas had very low T*β*4 expression on both the transcriptional and translational levels, and the other tissues had moderate expression of T*β*4. In contrast, human pancreatic islet cells highly express T*β*4 in previous study [32]. In mouse islet cells, however, T*β*4 was not expressed as much as human islets in this study. This may be partly due to the different reactivity of antibodies against T*β*4 between mouse and human tissue. Peritoneal macrophages have high concentrations of T*β*4, and the spleen has various immune cells containing macrophages or macrophage-like cells [16], possibly explaining why it has the highest expression of T*β*4 among the various tissues. Additionally, it has been suggested that T*β*4's presence in other tissues may be related to the presence of macrophages or macrophage-like cells in those tissues [16]. T*β*4 expression is also significantly increased in the retinas of patients with proliferative diabetic retinopathy (PDR)[33]. The vitreous and plasma T*β*4 concentrations in the excised membranes of patients with PDR who underwent a pars plana vitrectomy (PPV) were significantly higher than those in the control group. The mRNA levels of both T*β*4 and VEGF were also significantly increased in the diabetic membranes compared to the nondiabetic membranes, and their increase appears to play a role in angiogenesis in the retina. In contrast, high-glucose concentrations in our study did not affect the* in vitro* transcriptional and translational expression of T*β*4 in splenocytes. This suggests that the T*β*4 expression level changed minimally in response to the change in glucose concentration.

The tissue repair activity of T*β*4 towards diabetic complications, such as neuropathy and nephropathy, does not appear to be related to the decreased T*β*4 expression in the serum and tissues of diabetic animals. Rather, T*β*4 seems to help tissue regeneration by inducing growth factors that are responsible for tissue repair. For instance, it induces insulin-like growth factor-1 (IGF-1), which modulates cell survival, metabolism, and glucose homeostasis in high glucose- (HG-) human umbilical vein endothelial cells (HUVECs) [34]. Also, T*β*4 is a chemotactic factor to migrate satellite cell-derived myoblasts and myocytes into muscle injury site [35]. Muscle damage promotes the local production of T*β*4, which chemoattracts myoblasts to regenerate muscle [36]. In addition, T*β*4 reverses the decreased expression of angiopoiein-1 (Ang1) in endothelial and Schwan cells in diabetic sciatic nerves under high-glucose concentrations through the PI3K/Akt signaling pathway [14]. Additionally, T*β*4 improves glucose intolerance and ameliorates insulin resistance in mice by acting as an insulin sensitizer through the increased phosphorylation of Akt signaling level [37].

## 5. Conclusions

T*β*4 expression levels in the plasma and tissues of diabetic mice were not changed compared to the control mice, while the T*β*4 level was known to change significantly according to disease state such as tumor and inflammatory disease. Also, hyperglycemia seemed not to modulate the expression of T*β*4 in STZ-induced diabetic mice. Thus, all these results indirectly suggest that the molecular mechanism by which T*β*4 treatment alleviates hyperglycemia-induced tissue damage in diabetic mice may not be due to replenishing T*β*4 levels that may have been decreased under hyperglycemic conditions. Rather, the diverse biological activities of T*β*4, such as regenerative and angiogenetic activities, may contribute to tissue repair by inducing growth factors, promoting cell migration and wound healing, exerting anti-inflammatory effects, and inhibiting apoptosis.

## Data Availability

The data used to support the findings of this study are available from the corresponding author upon request.

## References

[1] Sosne G., Qiu P., Goldstein A. L., Wheater M. (2010). Biological activities of thymosin *β*4 defined by active sites in short peptide sequences. *The FASEB Journal*.

[2] Sanders M. C., Goldstein A. L., Wang Y. L. (1992). Thymosin beta 4 (Fx peptide) is a potent regulator of actin polymerization in living cells.. *Proceedings of the National Acadamy of Sciences of the United States of America*.

[3] Malinda K. M., Sidhu G. S., Mani H. (1999). Thymosin *β*4 accelerates wound healing. *Journal of Investigative Dermatology*.

[4] Cha H. J., Jeong M. J., Kleinman H. K. (2003). Role of thymosin beta4 in tumor metastasis and angiogenesis. *JNCI: Journal of the National Cancer Institute*.

[5] Piao Z., Hong C., Jung M., Choi C., Park Y. (2014). Thymosin *β*4 induces invasion and migration of human colorectal cancer cells through the ILK/AKT/*β*-catenin signaling pathway. *Biochemical and Biophysical Research Communications*.

[6] Philp D., St-Surin S., Cha H.-J., Moon H.-S., Kleinman H. K., Elkin M. (2007). Thymosin beta 4 induces hair growth via stem cell migration and differentiation. *Annals of the New York Academy of Sciences*.

[7] Lee S. Y., Park M. J., Lee H. K. (2017). Increased Expression of Thymosin *β*4 Is Independently Correlated with Hypoxia Inducible Factor-1*α* (HIF-1*α*) and Worse Clinical Outcome in Human Colorectal Cancer. *Journal of Pathology and Translational Medicine*.

[8] Xiao Y., Chen Y., Wen J., Yan W., Zhou K., Cai W. (2012). Thymosin *β*4: A Potential Molecular Target for Tumor Therapy. *Critical Reviews in Eukaryotic Gene Expression*.

[9] Vasilopoulou E., Kolatsi-Joannou M., Lindenmeyer M. T. (2016). Loss of endogenous thymosin *β*4 accelerates glomerular disease. *Kidney International*.

[10] Tan W. K., Purnamawati K., Pakkiri L. S. (2018). Sources of variability in quantifying circulating thymosin beta-4: literature review and recommendations. *Expert Opinion on Biological Therapy*.

[11] Zhang Y., Zhang Z. G., Chopp M. (2017). Treatment of traumatic brain injury in rats with N-acetyl-seryl-aspartyl-lysyl-proline. *Journal of Neurosurgery*.

[12] Kleinman H., Sosne G. (2016). Thymosin *β*4 Promotes Dermal Healing. *Thymosins*.

[13] Zhu J., Su L., Zhou Y., Ye L., Lee K., Ma J. (2015). Thymosin *β*4 Attenuates Early Diabetic Nephropathy in a Mouse Model of Type 2 Diabetes Mellitus. *American Journal of Therapeutics*.

[14] Wang L., Chopp M., Szalad A. (2012). Thymosin *β*4 promotes the recovery of peripheral neuropathy in type II diabetic mice. *Neurobiology of Disease*.

[15] Wang L., Chopp M., Jia L. (2015). Therapeutic benefit of extended thymosin *β*4 treatment is independent of blood glucose level in mice with diabetic peripheral neuropathy. *Journal of Diabetes Research*.

[16] Hannappel E., Xu G. J., Morgan J., Hempstead J., Horecker B. L. (1982). Thymosin beta 4: a ubiquitous peptide in rat and mouse tissues.. *Proceedings of the National Acadamy of Sciences of the United States of America*.

[17] Tabata T., Kinoshita Y., Fujii S. (1989). Protection by thymosin fraction 5 from streptozotocin-induced diabetes in mice. *Cell Mol Biol*.

[18] Wang S. H., Chen G., Zhang K. (1992). Effects of thymosin and insulin on suppressor T cell in type 1 diabetes. *Diabetes Res 19(1*.

[19] Park E. K., Ryu M. H., Kim Y. H. (2006). Anti-inflammatory effects of an ethanolic extract from Clematis mandshurica Rupr. *Journal of Ethnopharmacology*.

[20] Lee S. Y., Kang J. M., Kim D. J. (2017). PGC1alpha Activators Mitigate Diabetic Tubulopathy by Improving Mitochondrial Dynamics and Quality Control. *Journal of Diabetes Research*.

[21] Song R., Choi H. M., Yang H., Yoo M. C., Park Y., Kim K. S. (2012). Association between serum thymosin *β*4 levels of rheumatoid arthritis patients and disease activity and response to therapy. *Clinical Rheumatology*.

[22] Choi H. M., Lee Y., Yang H., Yoo M. C., Kim K. S. (2011). Increased levels of thymosin *β*4 in synovial fluid of patients with rheumatoid arthritis: association of thymosin *β*4 with other factors that are involved in inflammation and bone erosion in joints. *International Journal of Rheumatic Diseases*.

[23] Mutchnick M. G., Lee H. H., Hollander D. I., Haynes G. D., Chua D. C. (1988). Defective in vitro gamma interferon production and elevated serum immunoreactive thymosin *β*4 levels in patients with inflammatory bowel disease. *Clinical Immunology and Immunopathology*.

[24] Frohm M., Gunne H., Bergman A.-C. (1996). Biochemical and antibacterial analysis of human wound and blister fluid. *European Journal of Biochemistry*.

[25] Lunin S. M., Novoselova E. G. (2010). Thymus hormones as prospective anti-inflammatory agents. *Expert Opinion on Therapeutic Targets*.

[26] Bollini S., Riley P. R., Smart N. (2015). Thymosin beta4: multiple functions in protection, repair and regeneration of the mammalian heart. *Expert Opin Biol Ther 15*.

[27] Han T. (2010). Serum thymosin *β*4 levels in patients with hepatitis B virus-related liver failure. *World Journal of Gastroenterology*.

[28] Roman-Pintos L. M., Villegas-Rivera G., Rodriguez-Carrizalez A. D., Miranda-Diaz A. G., Cardona-Munoz E. G. (2016). Diabetic Polyneuropathy in Type 2 Diabetes Mellitus: Inflammation, Oxidative Stress, and Mitochondrial Function. *Journal of Diabetes Research*.

[29] Aronson D. (2008). Hyperglycemia and the pathobiology of diabetic complications. *Advances in Cardiology*.

[30] Kaur H., Mutus B. (2012). Platelet function and thymosin beta4. *The Journal of Biological Chemistry*.

[31] Goodall G. J., Hempstead J. L., Morgan J. I. (1983). Production and characterization of antibodies to thymosin beta 4. *The Journal of Immunology*.

[32] Nemolato S., Cabras T., Cau F. (2010). Different thymosin beta 4 immunoreactivity in foetal and adult gastrointestinal tract. *PLoS ONE*.

[33] Wang J., Lu Q., Tao Y., Jiang Y., Jonas J. B. (2011). Intraocular expression of thymosin *β*4 in proliferative diabetic retinopathy. *Acta Ophthalmologica*.

[34] Kim S., Kwon J. (2015). Effect of thymosin beta 4 in the presence of up-regulation of the insulin-like growth factor-1 signaling pathway on high-glucose-exposed vascular endothelial cells. *Molecular and Cellular Endocrinology*.

[35] Hara T. (2011). Thymosins and Muscle Regeneration. *Vitamins & Hormones*.

[36] Tokura Y., Nakayama Y., Fukada S.-I. (2011). Muscle injury-induced thymosin *β*4 acts as a chemoattractant for myoblasts. *The Journal of Biochemistry*.

[37] Zhu J., Su L.-P., Ye L., Lee K.-O., Ma J.-H. (2012). Thymosin beta 4 ameliorates hyperglycemia and improves insulin resistance of KK Cg-Ay/J mouse. *Diabetes Research and Clinical Practice*.

